# Supplementary material for: Bullying victimization among in-school adolescents in Sierra Leone: A cross-sectional analysis of the 2017 Sierra Leone Global School-Based Health Survey
Source: PLOS Glob Public Health. 2023 Dec 22;3(12):e0002498. doi: 10.1371/journal.pgph.0002498 (PMC10745186; doi:10.1371/journal.pgph.0002498)
Supplement: S1 Checklist — (DOCX) [file pgph.0002498.s001.docx]

STROBE Statement—checklist of items that should be included in reports of observational studies.

|  | Item No. | Recommendation | Page  No. | Relevant text from manuscript |
| --- | --- | --- | --- | --- |
| **Title and abstract** | 1 | (*a*) Indicate the study’s design with a commonly used term in the title or the abstract | 2 | A cross-sectional Analysis of the 2017 Sierra Leone Global School-Based Health Survey. |
|  |  | (*b*) Provide in the abstract an informative and balanced summary of what was done and what was found | 1-2 | Our findings suggest that bullying is a widespread problem among Sierra Leonean school-aged youth, and alcohol drinking, loneliness, suicide attempt and school truancy are potential risk factors. |
| Introduction | | | |  |
| Background/rationale | 2 | Explain the scientific background and rationale for the investigation being reported | 2-4 | In Sierra Leone, no studies have examined the prevalence of bullying or its effects on its victims. |
| Objectives | 3 | State specific objectives, including any prespecified hypotheses | 2-4 | This research sought to determine bullying victimization prevalence and its associated factors among Sierra Leonean school-going adolescents.. |
| Methods | | | |  |
| Study design | 4 | Present key elements of study design early in the paper | 4-5 | The 2017 Global School Health Survey in Sierra Leone provided a cross-sectional dataset that we were able to utilize |
| Setting | 5 | Describe the setting, locations, and relevant dates, including periods of recruitment, exposure, follow-up, and data collection | 4-5 | The Sierra Leone data collection uses a two-stage cluster sampling methodology to collect an accurate cross-section of the country's 10–19-year-old student population. The first step includes picking schools with a probability proportional to students' enrolment. The second stage is randomly picking classes such that every student has an equal chance of being selected. The response rates for schools in Sierra Leone's GSHS were 94%, and the student response rate was 87% [5]. |
| Participants | 6 | (*a*) *Cohort study*—Give the eligibility criteria, and the sources and methods of selection of participants. Describe methods of follow-up  *Case-control study*—Give the eligibility criteria, and the sources and methods of case ascertainment and control selection. Give the rationale for the choice of cases and controls  *Cross-sectional study*—Give the eligibility criteria, and the sources and methods of selection of participants | 4-5 | The Sierra Leone data collection uses a two-stage cluster sampling methodology to collect an accurate cross-section of the country's 10–19-year-old student population. The first step includes picking schools with a probability proportional to students' enrolment. The second stage is randomly picking classes such that every student has an equal chance of being selected. The response rates for schools in Sierra Leone's GSHS were 94%, and the student response rate was 87% [5]. |
|  |  | (*b*) *Cohort study*—For matched studies, give matching criteria and number of exposed and unexposed  *Case-control study*—For matched studies, give matching criteria and the number of controls per case |  |  |
| Variables | 7 | Clearly define all outcomes, exposures, predictors, potential confounders, and effect modifiers. Give diagnostic criteria, if applicable | 5 | The study's dependent variable was bullying victimization. |
| Data sources/ measurement | 8* | For each variable of interest, give sources of data and details of methods of assessment (measurement). Describe comparability of assessment methods if there is more than one group | *4* | The 2017 Global School Health Survey in Sierra Leone. |
| Bias | 9 | Describe any efforts to address potential sources of bias | 9-10 | We used the complex sampling command on SPSS to account for weighting and complex sampling design. |
| Study size | 10 | Explain how the study size was arrived at | 4-5 | The Sierra Leone data collection uses a two-stage cluster sampling methodology to collect an accurate cross-section of the country's 10–19-year-old student population. The first step includes picking schools with a probability proportional to students' enrolment. The second stage is randomly picking classes such that every student has an equal chance of being selected. |

Continued on next page

| Quantitative variables | 11 | Explain how quantitative variables were handled in the analyses. If applicable, describe which groupings were chosen and why | 5-9 | Age, gender, grade, truancy, alcohol usage, suicidal thoughts, suicide attempt, loneliness, anxiety, and being bullied were some demographic and health risk characteristics examined. The protective factors are (peer support, close friends, parental or guardian supervision, parental or guardian bonding, and parental or guardian connectedness) |
| --- | --- | --- | --- | --- |
| Statistical methods | 12 | (*a*) Describe all statistical methods, including those used to control for confounding | 10 | Descriptive, Pearson chi-square and binary logistic regression analyses were carried out. |
|  |  | (*b*) Describe any methods used to examine subgroups and interactions | 10 | We used the complex sampling command on SPSS to account for weighting and complex sampling design. All variables were included in the logistic regression, but only those with a significant connection (p 0.05) were used. The regression analysis findings were shown as odds ratios (aOR) with 95% confidence intervals (CIs). |
|  |  | (*c*) Explain how missing data were addressed | 10 | Listwise deletion was used to address missing data. |
|  |  | (*d*) *Cohort study*—If applicable, explain how loss to follow-up was addressed  *Case-control study*—If applicable, explain how matching of cases and controls was addressed  *Cross-sectional study*—If applicable, describe analytical methods taking account of sampling strategy |  |  |
|  |  | (*e*) Describe any sensitivity analyses Not Applicable |  |  |
| Results | | | | |
| Participants | 13* | (a) Report numbers of individuals at each stage of study—eg numbers potentially eligible, examined for eligibility, confirmed eligible, included in the study, completing follow-up, and analysed | Not applicable |  |
|  |  | (b) Give reasons for non-participation at each stage | Not applicable |  |
|  |  | (c) Consider use of a flow diagram | Not applicable |  |
| Descriptive data | 14* | (a) Give characteristics of study participants (eg demographic, clinical, social) and information on exposures and potential confounders | 10-12 | The percentage of Sierra Leonean adolescents who had been bullied while attending school was 48.7%. |
|  |  | (b) Indicate number of participants with missing data for each variable of interest | Not applicable |  |
|  |  | (c) *Cohort study*—Summarise follow-up time (eg, average and total amount) |  |  |
| Outcome data | 15* | *Cohort study*—Report numbers of outcome events or summary measures over time | Not applicable |  |
|  |  | *Case-control study—*Report numbers in each exposure category, or summary measures of exposure |  |  |
|  |  | *Cross-sectional study—*Report numbers of outcome events or summary measures | *Table 2 and figure1* |  |
| Main results | 16 | (*a*) Give unadjusted estimates and, if applicable, confounder-adjusted estimates and their precision (eg, 95% confidence interval). Make clear which confounders were adjusted for and why they were included | 10-14 | Table 3 |
|  |  | (*b*) Report category boundaries when continuous variables were categorized | Not applicable |  |
|  |  | (*c*) If relevant, consider translating estimates of relative risk into absolute risk for a meaningful time period | Not applicable |  |

Continued on next page

| Other analyses | 17 | Report other analyses done—eg analyses of subgroups and interactions, and sensitivity analyses |  |  |
| --- | --- | --- | --- | --- |
| Discussion | | | | |
| Key results | 18 | Summarise key results with reference to study objectives | 14-16 | Adolescents in Sierra Leone often fall subject to bullying, which has been associated with increased rates of alcohol use, isolation, suicidality, and truancy. This study lends credence to the idea that bullying is a widespread problem among Sierra Leonean school-aged youth. |
| Limitations | 19 | Discuss limitations of the study, taking into account sources of potential bias or imprecision. Discuss both direction and magnitude of any potential bias | 16 | Only teenagers who were school going and physically present on the day the data was conducted were considered so our findings are not a representative of all adolescents in Sierra Leone. |
| Interpretation | 20 | Give a cautious overall interpretation of results considering objectives, limitations, multiplicity of analyses, results from similar studies, and other relevant evidence | 16 | This study lends credence to the idea that bullying is a widespread problem among Sierra Leonean school-aged youth. |
| Generalisability | 21 | Discuss the generalisability (external validity) of the study results | 16 | This research is the first to use nationally representative data to assess the prevalence of bullying victimization among school-going teenagers aged 11-18 years in Sierra Leone. |
| Other information | |  | | |
| Funding | 22 | Give the source of funding and the role of the funders for the present study and, if applicable, for the original study on which the present article is based | 17 | Not funded |
|  |  |  |  |  |

*Give information separately for cases and controls in case-control studies and, if applicable, for exposed and unexposed groups in cohort and cross-sectional studies.

**Note:** An Explanation and Elaboration article discusses each checklist item and gives methodological background and published examples of transparent reporting. The STROBE checklist is best used in conjunction with this article (freely available on the Web sites of PLoS Medicine at http://www.plosmedicine.org/, Annals of Internal Medicine at http://www.annals.org/, and Epidemiology at http://www.epidem.com/). Information on the STROBE Initiative is available at www.strobe-statement.org.
