# Supplementary material for: Bullying victimization among in-school adolescents in Sierra Leone: A cross-sectional analysis of the 2017 Sierra Leone Global School-Based Health Survey
Source: PLOS Glob Public Health. 2023 Dec 22;3(12):e0002498. doi: 10.1371/journal.pgph.0002498 (PMC10745186; doi:10.1371/journal.pgph.0002498)
Supplement: Attachment — Submitted filename: Response to Reviewers.docx [file pgph.0002498.s002.docx]

The Editor

PLOS Global Public Health

25^th^ September 2023

**Ref: Submission ID PGPH-D-23-01085 - EMID:18c587c533bd8ba1**

Title: Bullying Victimization among in-School Adolescents in Sierra Leone: A cross-sectional Analysis of the 2017 Sierra Leone Global School-Based Health Survey.

Response to Reviewers' comments

Dear Sir/Madam,

We want to express our sincere thanks for painstakingly reviewing our manuscript and providing valuable comments and suggestions. Please see our point-by-point response to the reviewers' comments and suggestions. Revisions are highlighted in Red in the revised manuscript.

**Reviewers Comments**

**Reviewer 1**

**Reviewer 1 Comment 1:** The authors may consider reviewing the language of the article. At certain places the sentence structuring can be better to derive proper meaning.

**Authors' Response:** We have made the requested change. The language of the article is now clear and concise.

**Reviewer 1 Comment 2:** The Ethics review for secondary data analysis has been skipped. a waiver should have been obtained from Institutional ethics committee.
**Authors' Response:** Thanks for pointing this out. We used publicly available dataset hosted by the World Health Organization and the US Centers for Disease Control and Prevention and does not include any personally identifiable information. As such, no formal ethical approval to conduct this study was necessary. However, ethics approval was obtained from the Ministry of Health and Sanitation in Sierra Leone before conducting the survey in Sierra Leone.

**Reviewer 1 Comment 3:** In the first half of the paper there is inadequate clarity on temporality - thus it seems to suggest that the potential effects of bullying are rather the risk factors / predisposing factors for bullying - e.g., Alcohol use. This needs correction in language and sentenceframing.

**Authors ‘Response: Thanks for pointing that out. We have revised that portion of the introduction. It now reads.**

*“Understanding the risk factors contributing to bullying victimization among in-school adolescents in Sierra Leone is important for developing effective interventions to address the issue. One study. A Ghanian study found that students engaging in physical fights, being attacked physically, are injured, lonely, attempted suicide, and were current users of marijuana associated with bullying victimization among in-school adolescents [5]. These findings suggest that interventions to address bullying victimization need to consider the broader social and cultural context in which it occurs and should involve families, communities, and schools [5]. In addition, to the negative effects on mental health, research has indicated that adolescents who skip school or drop out are more likely to become victims of bullying and this can have long-term consequences for their future opportunities and well-being [6]. Addressing bullying victimization in schools is therefore important for young people's mental health, academic success, and prospects.”*

**Reviewer 1 Comment 4:** At many places only those factors which were statistically significant at 5% alpha error have been reported creating a kind of reporting bias. e.g., lack of association with suicidal ideation but presence of statistically significant association with suicidal attempt needs reporting and explanation, including limitation.

**Authors' Response:** We have added the requested change. The sentence now reads ***“****Bullying victimization among Sierra Leonean adolescents in school is shown in (Table 2), along with a bivariate analysis. The percentage of Sierra Leonean- school-going adolescents bullied while attending school was 48.7%. Victimization by bullies varied greatly by factors such as loneliness, attempts at suicide, substance abuse, and school absences. Significant differences across variables all had p-values of less than 0.05. Males accounted for the largest proportion of bullying victims (53.1%), lonely school-aged children (23%), and those who had attempted suicide (24.5%). Adolescents who regularly partake in the usage of both alcohol (19.2%) and cannabis (6.3%) are disproportionately likely to be bullied while at school. Also, students who skipped class were likelier to become bully victims than those who stayed in class (41.1%). The variables that did not show statistically significant associations with bullying victimization include age, sex, grade, anxiety, suicidal ideation, suicidal plan, close friends, peer support, and parental support, with p-values greater than 0.05”.*

“*The results of the multivariate logistic regression are shown in (Table 3). Adolescents who drank alcohol [aOR=****2.48,*** *95% CI =* ***1.50-4.10****] were more likely to be bullied. Furthermore, teenagers who reported feelings of loneliness [aOR=* ***1.51,*** *95% CI =* ***1.07-2.14]*** *were more likely to report being bullied than their less lonely peers. Adolescents who had already attempted suicide [aOR=* ***1.72,*** *95% CI =* ***1.03-2.87]*** *were also more vulnerable to being bullied. Similarly, school truancy [aOR=* ***1.53,*** *95% CI =* ***1.24-1.88****] among teenagers was associated with an increased risk of being bullied. The variables that did not show statistically significant associations with bullying victimization include age, sex, grade, cannabis use, anxiety, suicidal ideation, suicidal plan, close friends, peer support, and parental support”.*

**Reviewer 1 Comment 5:** Very high levels of suicidal attempt have been reported. However, the same has not received much attention in conclusion and recommendation to authorities.

**Authors' Response:** We have added the requested change. The sentence now reads *“Our study reported an alarmingly high levels of suicidal attempt among adolescents in Sierra Leone. This study also lends credence to the idea that bullying is a widespread problem among Sierra Leonean school-aged youth. Increased rates of alcohol use, loneliness, suicidal attempt and truancy were associated with being bullied. In light of the aforementioned risk factors of bullying in schools, policymakers and school administrators in Sierra Leone need to develop and execute anti-bullying policies and initiatives that target the underlying causes of bullying among teenagers. They should also increase awareness on suicide in Sierra Leone. This can be done through public campaigns, school campaign and community outreach .”*

**Reviewer 1 Comment 6:** Figure needs proper labelling and is creating confusion with putting frequency and percentage in a single figure. consider revising.

**Authors' Response:** Thanks for pointing this out. We have revised the figure into a simple pie chart.

**Reviewer2**
**Reviewer 2 Comment 1:** Introduction: The introductory lines " Bullying victimization among in-school adolescents is a serious public health issue that has gained increasing attention in recent years." It is better to cite sources which provide data on bullying among adolescents rather than research articles. Description of school health services addressing adolescent health in Sierra Leone region has to be mentioned in the introductory section in order to provide overview.
**Authors' Response:** Thanks for pointing this out. We have added the following to the introductory line **“***Approximately 32% of adolescents have experienced peer bullying at school on one or many occasions. The Middle East, North Africa, and sub-Saharan Africa exhibit the highest prevalence of adolescents reporting instances of bullying, whereas Central America, the Caribbean, and Europe demonstrate the lowest rates.”*

Also we have added the Sierra Leone school health services in addressing adolescent health as  **“***Child and adolescent mental health in Sierra Leone has received limited attention, but there have been endeavours to enhance child and adolescent mental healthcare in a low resource country. The school's curriculum incorporates a comprehensive approach to health education. The curriculum encompasses various subjects, including sexual and reproductive health, hygienic practises, nutritional education, substance misuse prevention, and mental health awareness. Acknowledging the significance of mental health, school health services integrate counselling and support provisions aimed at assisting adolescents in managing stress, anxiety, and other mental health difficulties. Schools often coordinate and implement routine health campaigns and awareness programmes. These campaigns aim to tackle concerns such as HIV/AIDS prevention, malaria control, and vaccine initiatives, with the goal of equipping adolescents with comprehensive knowledge regarding preventive measures. [5****]”***

**Reviewer 2 Comment 2:** Methodology: In the explanatory variable, a detailed description of GSHS datasethastobeprovided.
**Authors' Response:** Thanks for pointing this out. A detailed description of the GSHS dataset and their recode was done in Table 1 of the article. Also, we have added the following texts in method section *under “sample and procedure.”*

*“The GSHS dataset provides a nationally representative information on health behaviours and protective factors that are predisposing factor morbidity and mortality among adolescents in middle- and lower-income countries. The goal is to support school health and youth health programs and policies worldwide [9]. GSHS collects data on the following health behaviours - alcohol use, tobacco use, dietary behaviours, drug use, hygiene, mental health and physical activity and violence and unintentional injury. Also, collects data on sexual behaviours such as HIV infection, other sexually transmitted infections, and unintended pregnancy. Protective factors include peer and parental support [9]”*

**Reviewer 2 Comment 3:** Results: Results section to provide data on total number of adolescents included in the analysis.
**Authors' Response:** We have added the requested change. ***“****A total of 2,798 in-school adolescents in Sierra Leone was analyzed in the present study”.* **Reviewer 2 Comment 4:** Discussion: In the limitation section, the authors commented on various limitations, however these were not potrayed in methodology section. Hence, sampling strategy has to be revised and provide detailed description rather than few lines.

**Authors' Response:** Thanks for pointing out. We have rewritten our limitations focusing on those portrayed in the methodology section. The limitation now reads “*The research conducted in Sierra Leone in 2017 employed a cross-sectional study design, which imposes constraints on our capacity to establish causal relationships between the dependent and explanatory variables. Additionally, it is crucial to acknowledge that the scope of our findings is restricted to teenagers who are enrolled in schools within Sierra Leone. Future study should place a higher emphasis on investigating the experiences and behaviour exhibited by adolescents in both educational and non-educational environments. The presence of recall bias in the data is a potential concern as it is influenced by the dependence on self-reported responses.”*

We hope that we have adequately addressed the reviewers' comments, and we look forward to receiving a favorable outcome on our paper.

Yours Sincerely,

Augustus Osborne

Corresponding Author
